# A simple and rapid method for combining fluorescent in situ RNA hybridization (FISH) and immunofluorescence in the *C. elegans* germline

**DOI:** 10.1016/j.mex.2016.05.001

**Published:** 2016-05-06

**Authors:** Dong Suk Yoon, DeQwon L. Pendergrass, Myon-Hee Lee

**Affiliations:** a Department of Medicine, Brody School of Medicine at East Carolina University, NC 27834, USA; b Lineberger Comprehensive Cancer Center, University of North Carolina, Chapel Hill, NC 27599, USA

**Keywords:** Fluorescent *in situ* hybridization (FISH), Immunofluorescence, *C. elegans*, Germline

## Abstract

Imaging of RNAs and proteins in specific tissues has opened ample avenues to understand gene expression during development. Recently, a fluorescent *in situ* RNA hybridization (FISH) method has been developed to analyze the spatio-temporal expression patterns of endogenous mRNAs. However, combining FISH with immunofluorescence is challenging as the reaction conditions for the two procedures conflict in multiple ways. In this report, we developed a simple and rapid method to detect both RNAs and associated proteins with better preservation of the fine structure in the *C. elegans* germline. This method will provide new tools for *in vivo* imaging of RNAs and their associated proteins in the same germline, which also enables simultaneous visualization of RNA/protein complex at the cellular level *in vivo*.

• Developing a simple and rapid FISH method with better preservation of the fine structure.
• Combining FISH with immunofluorescence in *C. elegans* germline.
• Labeling extruded gonads, instead of the whole worms, to prevent non-specific somatic autofluorescence.

## Graphical abstract

**Figure:**
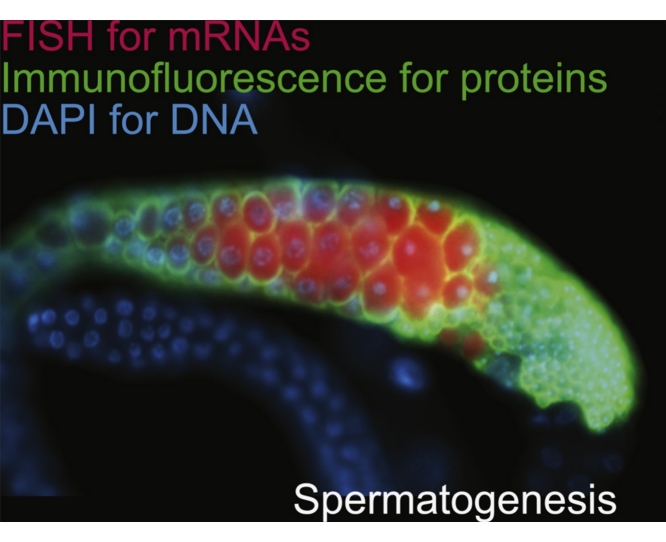

## Background

**Fig. 1:**
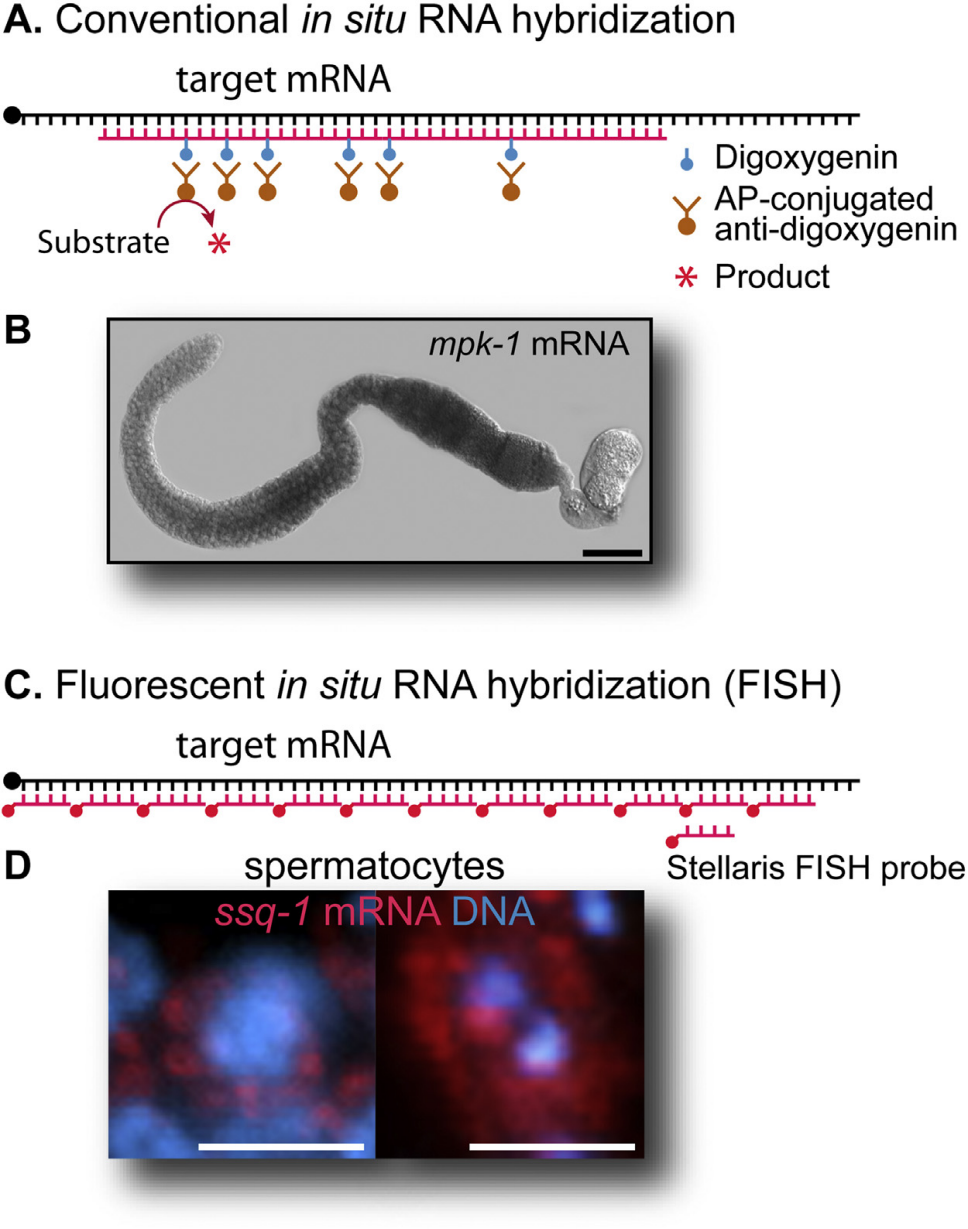
*in situ* mRNA detection methods. (A) Schematic of conventional *in situ* mRNA hybridization method. Digoxigenin-labeled RNA antisense probes (200–500 base pairs) are produced using PCR and *in vitro* transcription. To visualize the hybridized RNA probe, dissected gonads are incubated with alkaline phosphatase (AP)-conjugated anti-digoxigenin antibody (Roche) and then with the color-developing reagents, 4-nitroblue tetrazolium chloride and 5-bromo-4-chloro-3-indolyl-phosphate (NBT/BCIP). (B) The localization of *mpk-1* (an ERK homolog) mRNA in *C. elegans* gonad. Scale bar: 50 μm. (C) Schematic of FISH method. (D) The subcellular localization of *ssq-1* (sperm-specific family, class Q) mRNA in *C. elegans* spermatocytes. Primary spermatocyte (left) and secondary spermatocyte (right). Scale bar: 3 μm.

Tracking expression and subcellular localization of RNAs and proteins during development provides important clues to understand their biological and physiological function. In conventional *in situ* RNA hybridization methods, sliced tissues are incubated with biotin or digoxygenin-labeled RNA probes that are synthesized by PCR, and then endogenous RNAs:RNA probe hybrids are visualized by antibodies against biotin or digoxygenin (Fig. 1A and B). This two-step procedure exhibited low sensitivity and non-specific probe binding. Therefore, a highly sensitive RNA detection method was required to analyze the *in vivo* subcellular localization of endogenous RNAs with better preservation of the fine structure.

Recently, an advanced fluorescent *in situ* RNA hybridization (henceforth called “FISH”) method using fluorescent (e.g., Alexa 594, Alexa 488, Cy3, or Cy5)-labelled small RNA molecule (∼20 nucleotides) probes has been developed (Fig. 1C and D) in multiple organisms, including *C. elegans* whole worms and embryos [1], yeast [2], zebrafish embryos [3], and Drosophila egg chamber [4]. Particularly, this FISH method enables the analysis of several different RNAs simultaneously using multi-color fluorescence and the analysis of transcriptional activity at the cellular level. Although this method has made a significant progression in analyzing the expression pattern and subcellular localization of the specific RNAs, combining FISH with immunofluorescence (henceforth called “IF”) method to detect RNAs and their associated proteins simultaneously is a practically challenge due to different hybridization conditions (Saline-sodium citrate (SSC) for FISH; Phosphate-buffered saline (PBS) for IF), different reaction temperatures (≥30 °C, usually with formamide for FISH; 4–24 °C, no harsh chemicals for IF), and RNase sensitivity [5] (Table 1). To date, this combining method has been developed only in Drosophila egg chamber [4] and embryos [5]. Therefore, developing the FISH/IF combining protocol will lead to advanced biochemical, cellular, and functional analysis of RNA:protein complex in the specific cells or tissues.

The nematode *Caenorhabditis elegans* (*C. elegans*) is an attractive model organism for study of basic biological and biomedical sciences. Particularly, RNA regulations by RNA-binding factors (e.g., RNA-binding proteins and microRNAs) have been studied intensively in the *C. elegans* germline [6], [7]. These RNA regulations control germline stem cell (GSC) maintenance, mitosis/meiosis decision, and sex determination [8]. Aberrant regulations result in sterility and germline tumors [7]. Therefore, the simultaneous analysis of RNAs and RNA-binding proteins in the specific cell types and tissues provides a powerful tool for investigating its cellular and physiological function *in vivo*. To date, in *C. elegans*, FISH method has been developed in only whole worms and embryos [1], but not in germlines. Moreover, FISH/IF combining protocol has not yet been developed because the reaction conditions for the two procedures conflict in multiple ways (Table 1). We here present a simple protocol to visualize RNAs and proteins simultaneously using FISH and IF in the same *C. elegans* germline. This method enables detection of RNA/protein complex with better preservation of the fine structure at the cellular level.

## Method details

### Probe design and synthesis

Stellaris FISH probes were designed as described by Ji and van Oudenaarden [1]. Briefly, FISH probe length is 17–22 base pairs, while probe spacing is no less than 2 base pairs. Probe GC contents are about 45%, and the number of probes for each target RNA are between 30 and 96 depending on target transcript length. Typically, 48 probes are used to ensure good signal quality. A web-based probe design software is available at: http://www.biosearchtech.com/stellarisdesigner/

### *C. elegans* culture and synchronize worm

1. Worms are cultured on 100 mm NGM (Nematode Growth Media) agar plates seeded with OP50 *E. coli* bacteria at permissive temperature (20 °C), as described [9].

2. 4–5 days after plating, (when there are many adult worms on NGM agar plates), wash worms off NGM agar plates with 5 ml of M9 buffer per plate and transfer them into a 15 ml conical tube.

3. Pellet worms by table-top centrifugation at 1000 rpm for 1 min and carefully remove the supernatant.

4. Add 10 ml bleaching solution to the conical tube with the worm pellet.

5. Wait until over 90% of the worms are lysed and several embryos remain. (typically, this takes about 5–6 min).

**Note:** Vortexing every one minute helps the bleach lysis. Bleaching longer than 10 min will damage embryos.

6. Spin down the embryos at 1000 rpm for 1 min and remove the bleaching solution.

7. Wash three times with 10 ml of M9 buffer.

8. After the last centrifugation, add 10 ml of M9 solution to the tube with embryos and allow the embryos to hatch in 20 °C incubator by locking the tube gently.

9. About 12 h later, spin down worms at 1000 rpm for 1 min, remove the supernatant, and monitor hatched L1 worms by placing 1 drop of the samples on a slide glass and estimate the number of hatched L1 worms.

10. Plate about 200 L1 worms on an NGM agar plate seeded with OP50 *E. coli* bacteria.

11. Incubate them for three days at 20 °C.

### Germline dissection and fixation

12. Transfer about 50 adult worms to a glass dish using a platinum worm picker.

**Note:** Alternatively, wash worms off a plate with 1.5 ml of PTW/Levamisole and transfer to a glass dish (60 × 15 mm), filled 5 ml PTW/Levamisole solution.

**Fig. 2:**
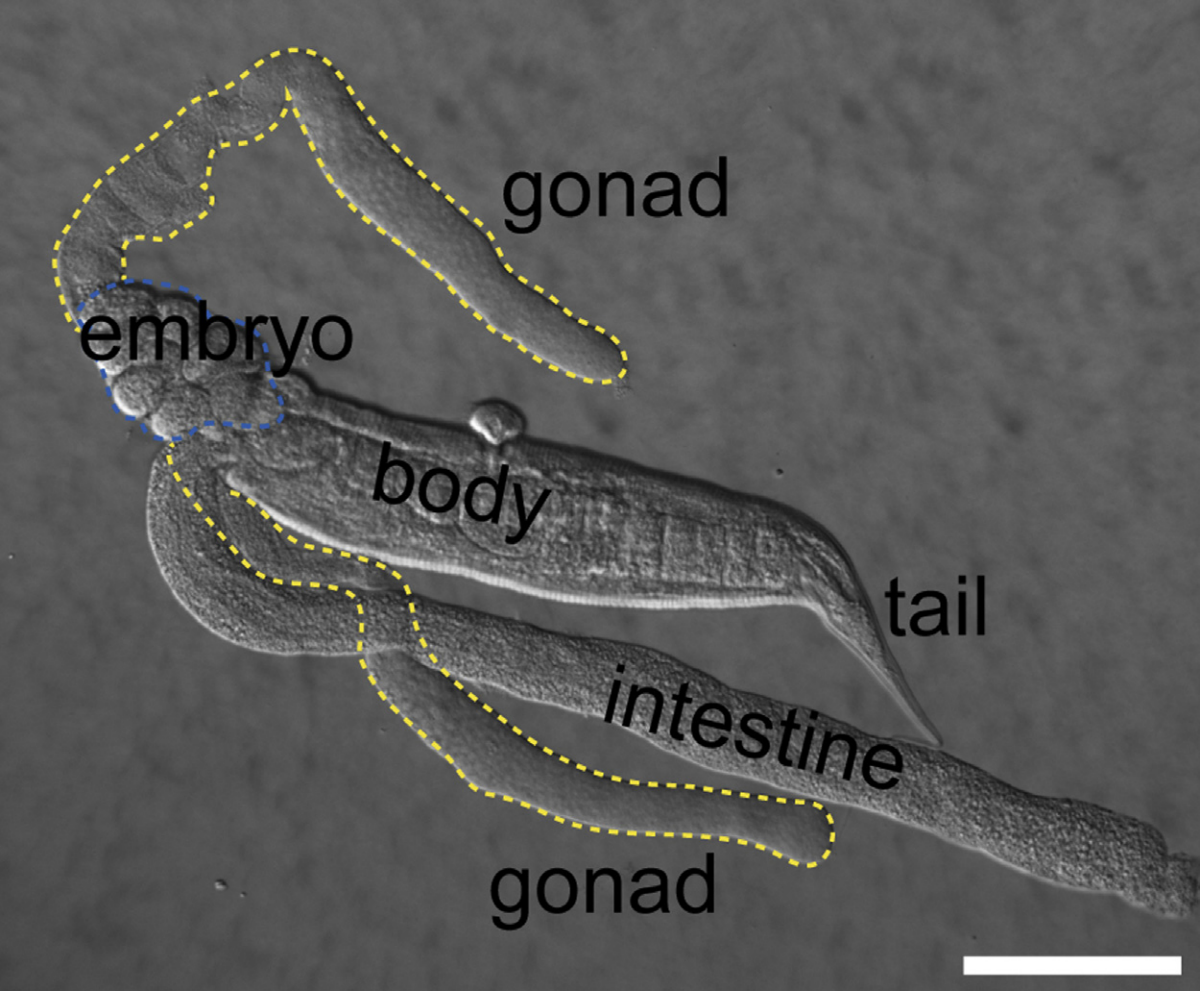
Gonad dissection. DIC picture of a decapitated adult *C. elegans* hermaphrodite. *C. elegans* has two gonadal arms. Scale bar: 100 μm.

13. Once worms are paralyzed (1–2 min), cut off head or tail parts using a disposable scalpel under a dissecting microscope (Fig. 2).

**Note:** Dissecting 50 gonads within 15 min will make better germline morphologies.

14. Transfer dissected worms to a 1.5 ml microcentrifuge tube.

15. Spin down at about 8000 rpm for 2–3 s using a mini-centrifuge and carefully remove the supernatant using a micropipette.

16. Add 200 μl of 3% paraformaldehyde (PFA) fixation solution to the microcentrifuge tube and incubate 10–30 min at room temperature.

**Note:** For tumorous germlines, 30-min fixation will help to make better germline morphologies.

17. Spin down the dissected worms at about 8000 rpm for 2–3 s using the mini-centrifuge and carefully remove the PFA solution with a 200 μl pipette tip to leave dissected worms.

18. Wash two times with PTW solution.

19. After the last centrifugation, carefully remove the PTW solution, add 200 μl of ice-cold 100% methanol to the microcentrifuge tube, and incubate for 10 min at −20 °C.

**Note:** Alternatively, fixed gonads can be stored in cold methanol at −20 °C for a few days.

### FISH: hybridization and washing

20. Add an equal volume of PTW solution (200 μl) with methanol to the microcentrifuge tube and immediately spin down to remove the supernatant.

21. Wash the dissected worms two times with PTW solution.

22. After the last centrifugation, add 100 μl of FISH washing solution to the microcentrifuge tube and let stand for 2–5 min.

**Note:** During incubation time, prepare Stellaris RNA probe hybridization solution (1 μl of RNA probe in 100 μl of hybridization solution).

23. Carefully remove the FISH washing solution after spinning down at about 8000 rpm for 2–3 s using a mini-centrifuge.

24. Add Stellaris RNA probe to the dissected worm and incubate them for 4 h at 37 °C or overnight at 30 °C.

25. Carefully remove the Stellaris RNA probe after spinning down at about 8000 rpm for 2–3 s using a mini-centrifuge and wash one more time with FISH washing solution.

26. After removing FISH washing solution after centrifugation, add 100 μl of FISH washing solution to the microcentrifuge tube and incubate for 30 min at 30 °C.

**Note:** Move to step 35 if immunofluorescence is not necessary.

### Immunofluorescence

27. Wash three times with PTW/BSA solution.

28. After the last centrifugation, add 200 μl of PTW/BSA solution to the microcentrifuge tube and incubate for 30 min.

**Note:** Longer incubation will reduce non-specific staining.

29. During incubation time, prepare primary antibodies of interest.

30. Add primary antibody to the microcentrifuge tube and incubate for 2 h at room temperature.

**Note:** Alternatively, incubate dissected gonads in primary antibody solution overnight at 4 °C.

31. Wash three times for at least 30 min (10-min interval) with PTW/BSA solution.

32. Prepare fluorescence-conjugated secondary antibodies, and then dilute the secondary antibodies in the PTW/BSA solution at a final concentration of 1:200.

33. Incubate the samples in the PTW/BSA solution containing the fluorescence-conjugated secondary antibodies for 1–2 h at room temperature.

**Note:** To distinguish FISH and IF signals, use secondary antibodies conjugated with different fluorochromes from that used for FISH probe (i.e., Cy3 (Red) for FISH and Alexa 488 (Green) for IF).

34. Carefully remove the supernatant by spinning down at about 8000 rpm for 2–3 s using a mini-centrifuge.

35. Add DAPI solution (100 ng/mL) to the microcentrifuge tube and incubate them for 10 min at room temperature.

36. Carefully remove the supernatant by spinning down about 8000 rpm for 2–3 min using a mini-centrifuge, and then wash three times using PTW/BSA solution for at least 30 min (10-min interval).

### Image acquisition

37. Completely dissolve 2% agar using a microwave.

38. Drop 2% agar liquid on the center of glass slide, and immediately place another glass slide on the glass slide with agar drop.

39. After gelation, carefully remove the upper glass slide, and then transfer the stained germline samples onto the agar pad on glass slide and immediately aspirate the residual liquid using a mouth pipette.

40. Drop 10 μl of antifade mounting solution (e.g., VECTASHIELD) onto the stained germline samples and carefully spread them to avoid the overlap of samples.

41. Carefully place a coverslip over the top to put the sample between the agar pad and coverslip.

**Note:** If necessary, seal the coverslip with nail polish to prevent evaporation of antifade mounting solution.

42. Take pictures using a fluorescence microscopy and analyze the results.

## Method validation

**Fig. 3:**
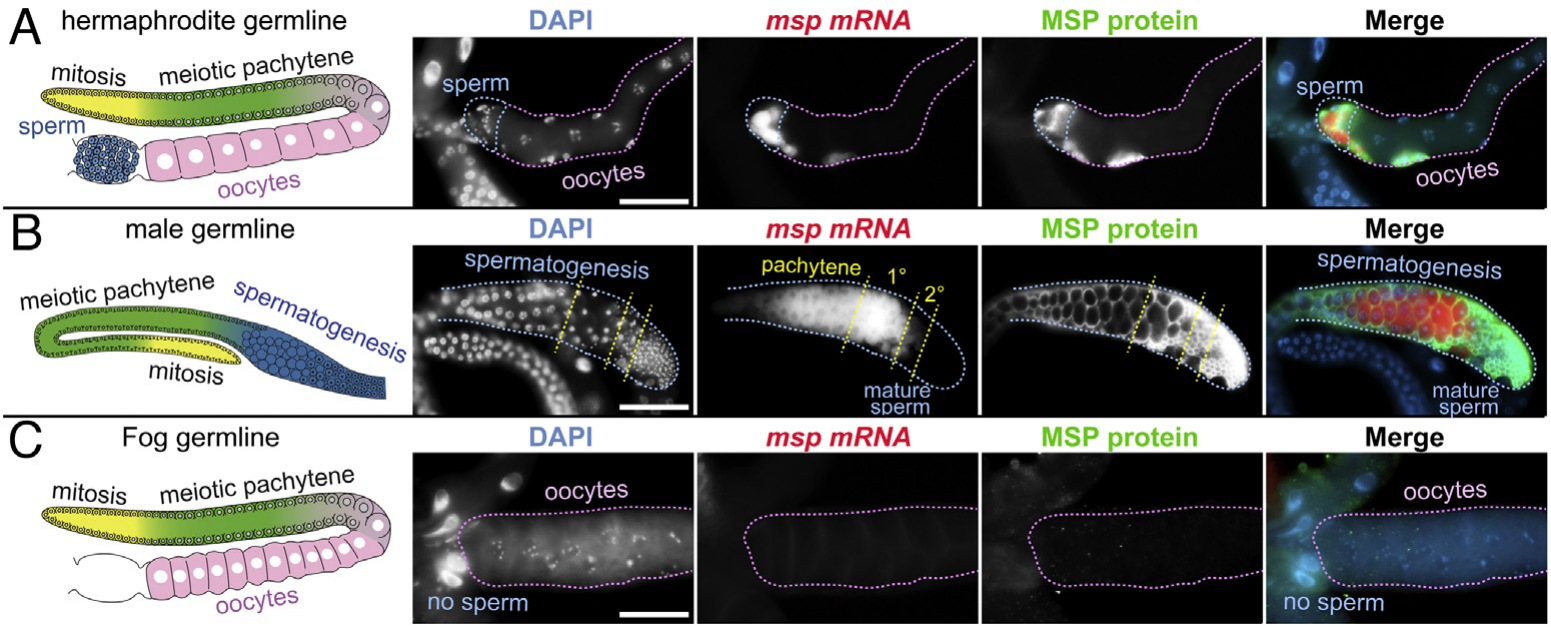
FISH and Immunofluorescence for *msp* RNAs and MSP proteins. Schematics (1^st^ column) of adult hermaphrodite (A), adult male (B), and adult *fog-3(q520)* mutant (C) germlines. Germline staining with DAPI (2^nd^ column), *msp* FISH probes (3^rd^ column), and anti-MSP antibodies (4^th^ column). Merges (5^th^ column) of DAPI (blue), *msp mRNAs* (red), and MSP proteins (green). ‘1°’ indicates “primary spermatocytes”; ‘2°’ indicates “secondary spermatocytes”. Scale bar: 50 μm.

In this report, we first developed a simple combining protocol by adapting the FISH labeling procedure from previous protocols [1] and by optimizing reaction conditions for both FISH and IF using *C. elegans* germline (Table 1). Current FISH and IF procedures usually take three or four days in *C. elegans* whole worms [1]. Furthermore, it has been generally accepted that combining RNA FISH with IF can be technically challenging because of RNase contamination in IF reaction solutions (especially crude antibodies). However, our combined protocol can be done in one or two days in the dissected gonads, depending on antibody sensitivity. In addition, tissue samples that were fixed with 3–4% paraformaldehyde and/or methanol retain more RNAs compared to other conventional fixatives, such as Carnoy’s solution (60% ethanol, 30% chloroform and 10% glacial acetic acid), and therefore show strong and stable FISH signals [10], [11], [12]. We used this approach to examine the expression of *msp* mRNAs and MSP proteins (Fig. 3). The MSPs (Major Sperm Proteins) are encoded in the *C. elegans* genome by a multigene family in small clusters at three chromosomal loci [13]. In spite of their dispersed locations, all of the MSP genes are expressed exclusively in the spermatogenic germline, but not in the oogenic germlines. This protocol was thus tested in three different germlines: wild-type hermaphrodite with both sperm and oocytes (Fig. 3A), wild-type male germlines with only spermatogenic germ cells (Fig. 3B), and *fog-3(q520)* mutant germlines [14] with only oogenic germ cells as a negative control (Fig. 3C). FOG-3 is critical for sperm fate specification [14], [15]. For FISH experiment, we used Stellar FISH probes for *msp* gene as previously described [1], [16]. For both FISH and IF, we tested three different fixation conditions: (i) 4% paraformaldehyde/1X PBS followed by 70% Ethanol, (ii) 3% paraformaldehyde/0.25% glutaraldehyde/0.1 M K_2_HPO_4_ (pH7.2), and (iii) 3% paraformaldehyde/0.1 M K_2_HPO_4_ (pH 7.2) followed by 100% Methanol. We found that only protocol (iii) reserved both signals: *msp* RNAs (Red in Fig. 3A and B) and MSP proteins (Green in Fig. 3A and B) were highly accumulated in both the late pachytene meiotic germ cells and primary (1°) spermatocytes (Fig. 3A and B). Notably, MSP proteins persisted in secondary (2°) spermatocytes and mature sperm (Fig. 3B), whereas *msp* mRNAs were dramatically decreased in secondary spermatocytes and were undetectable in mature sperm (Fig. 3B). We also obtained similar results with another RNA probes for sperm-specific gene, *ssq-1* (Sperm-Specific family, class Q) [16] (data not shown). Their specificities were determined in the feminized *fog-3(q520)* mutant germline: no *msp* RNAs and MSP proteins were detected.

We next tested two different protocols to find the better condition: (i) IF followed by FISH and (ii) FISH followed by IF. We found that protocol (i) resulted in a decrease of the IF signals, indicating that FISH hybridization condition (SSC buffer, higher hybridization temperature, and formamide chemical) may distort IF signals. However, protocol (ii) reserved both signals (Fig. 3A and B**)**. The advantage of this protocol is three-fold: (1) A simplified FISH protocol with better preservation of the fine structure, (2) Reduced non-specific somatic autofluorescence: FISH and IF in the extruded germlines eliminate autofluorescence signals from somatic tissues (e.g., intestine), and (3) Combined FISH and immunofluorescence in the same germline. Therefore, our combining protocol may significantly contribute to the study of RNA/protein regulation during *C. elegans* germline development. However, this FISH/IF combining protocol is limited to the *C. elegans* germline. Therefore, finding the simple combining labeling protocol in *C. elegans* whole worms and embryos will provide a benefit to better understand the mechanism of RNA/protein regulation in other tissues.

## Conflict of interests

The authors declare no conflict of interest.

## Figures and Tables

**Table 1:** Comparison of FISH and IF conditions.

Steps	Embryos	Whole worms	Germlines
FISH	1. Fix the samples in 4% paraformaldehyde in 1× PBS at room temperature for 45 min.	1. Fix the samples in 4% paraformaldehyde in 1× PBS at room temperature for 15 min.	1. Fix the samples in 3% paraformaldehyde in 0.1 M K_2_HPO_4_ at room temperature (RT) for 10 min.
	2. Post-fixation in 70% Ethanol for overnight (or longer).	2. Post-fixation in 70% Ethanol for overnight (or longer).	2. Post-fixation in 100% cold methanol for 10 min.
	3. Incubate the fixed whole worms with RNA probes at 37 °C for 4 h.		

IF	FISH/IF combining method is not applicable. Typical IF method for embryos is “embryo freeze-cracking and methanol/acetone fixation” (see http://www.wormatlas.org/EMmethods/Earlyembryostain.htm)	FISH/IF combining method is not applicable. Typical IF method for whole worms is “whole worm freeze-cracking” [13] or “ Antibody staining of formaldehyde-fixed whole worms” by Gary Ruvkun and Michael Finney (see http://www.wormatlas.org/images/finneyruvkun.pdf)	1. Incubate the samples with blocking solution (1XPBS + 0.1% Tween20 +0.5% BSA).
			2. Primary antibody incubation (2 h at RT).
			3. Washing with blocking solution for 30 min.
			4. Secondary antibody incubation (2 h at RT).
			5. Washing with blocking solution for 30 min.
			6. DAPI staining.
			7. Washing one more time.
			8. Mounting the samples onto slides with agar pad.
