# Supplementary material for: A simple and rapid method for combining fluorescent in situ RNA hybridization (FISH) and immunofluorescence in the C. elegans germline
Source: MethodsX. 2016 May 6;3:378–85. doi: 10.1016/j.mex.2016.05.001 (PMC4878318; doi:10.1016/j.mex.2016.05.001)
Supplement: Supplementary file 1 [file mmc1.pdf]

SUPPLEMENTARY MATERIALS FOR

A simple and rapid method for combining fluorescent in situ RNA hybridization (FISH) and immunofluorescence in the *C. elegans* germline

Dong Suk Yoon[a], DeQwon L. Pendergrass[a] and Myon-Hee Lee[a,b,*]

[a]Department of Medicine, Brody School of Medicine at East Carolina University, NC 27834, USA. [b]Lineberger Comprehensive Cancer Center, University of North Carolina, Chapel Hill, NC 27599, USA.

Reagents

Agar (Sigma-Aldrich, St. Louis, MO; Cat #: A7002-500g)
Agarose (VWR International, West Chester, PA; Cat #: BDH4098)
Antibodies for anti-MSP (Developmental Studies Hybridoma Bank, Iowa City, Iowa; Cat #: 4A5)
Antifade mounting solution-VECTASHIELD (Vector Laboratories, Burlingame, CA; Cat #: H-100)
Bovine Serum Albumin (BSA) (Sigma-Aldrich, St. Louis, MO; Cat #: A9418)
Caenorhabditis elegans strains (Caenorhabditis Genetics Center (CGC): http://cbs.umn.edu/cgc/home)
Conical tubes (15ml) (VWR International, West Chester, PA; Cat #: 89039-664)
Coverslips (VWR International, West Chester, PA; Cat #: 48366-227)
DAPI (4',6-diamidino-2-phenylindole) (Thermo Fisher, Waltham, MA; Cat # D1306)
Disposable scalpel (Feather Safety Razor, Kita-Ku, Osaka, Japan; Cat #: 2975)
***E. coli* strain OP50** (Caenorhabditis Genetics Center (CGC): http://cbs.umn.edu/cgc/home)
Glass petri dish (Carolina Biological Supply, Burlington, NC; Cat #: 721132)
Goat anti-Mouse IgG (H+L) Secondary Antibody, Cy3 conjugate (Thermo Fisher, Waltham, MA; Cat #: A10521)
Levamisole (Sigma-Aldrich, St. Louis, MO; Cat #: 196142)
Methanol (VWR International, West Chester, PA; Cat #: BDH1135)
Microcapillary Mouth pipette (Sigma-Aldrich, St. Louis, MO; Cat #: A5177)
Paraformaldehyde aqueous solution (16%), EM Grade (Electron Microscopy Sciences, Hatfield, PA; Cat #: 15700)
Phosphate-Buffered Saline (PBS) (Amresco, Solon, OH; Cat #: E703)
Petri dish (VWR International, West Chester, PA; Cat #: 25384-164)
Platinum worm picker (Tritech Research, Los Angeles, CA; Cat #: TWPH1)
Potassium phosphate dibasic (K_2HPO_4) (Sigma-Aldrich, St. Louis, MO; Cat #: P3786)
Slide glass (VWR International, West Chester, PA; Cat #: 490013-182)

Stellaris FISH hybridization buffer (Biosearch Technologies, Novato, CA; Cat #: SMF-HB1-10)
Stellaris FISH wash buffer (Biosearch Technologies, Novato, CA; Cat #: SMF-WB1-20)
Stellaris target RNA FISH probes (Biosearch Technologies, Novato, CA; Cat #: SMF-WB1-20)

Recipes

Bleaching Solution (25 mL)

Component	Volume	[final]
10 N Sodium hydroxide (NaOH)	1.25 mL	0.5 N
Sodium hypochlorite (Household bleach)	5.0 mL	1:5 dilution
Sterile H_2O	18.75 mL	
**Total**	**25 mL**	

LB (Luria Bertani) Broth (1L)

Component	Volume	[final]
Bacto-tryptone	10 g	10g/L
Bacto-yeast extract	5 g	5g/L
Sodium chloride (NaCl)	10 g	10g/L
H_2O	Up to 1,000 mL	
**Total**	**1,000**	

M9 Buffer (1L)

Component	Volume	[final]
Potassium phosphate monobasic (KH_2PO_4)	3 g	3g/L
Disodium phosphate (Na_2HPO_4)	6 g	6g/L
Sodium chloride (NaCl)	5 g	5g/L
1 M Magnesium sulfate ($MgSO_4$)	1 mL	1 mM
H_2O	999 mL	
**Total**	**1,000**	

NGM (Nematode Growth Media) Agar Plates (1L)

Component	Volume	[final]
Sodium chloride (NaCl)	3 g	3g/L
Peptone	2.5 g	2.5g/L
Agar	17 g	17g/L
1 M Calcium chloride ($CaCl_2$)	1 mL	1 mM
1 M Magnesium sulfate ($MgSO_4$)	1 mL	1 mM
1M Potassium Phosphate (KPO_4) (pH 6.0)	25 mL	25 mM
Cholesterol (5 mg/1mL in Ethanol)	1 mL	5 µg/mL
H_2O	975 mL	
**Total**	**1,000**	

3% Paraformaldehyde Fixation Solution (50 mL)

Component	Volume	[final]
16% Paraformaldehyde	9.375 mL	3 %
1M Potassium phosphate dibasic (K_2HPO_4, pH 7.2)	5 mL	100 mM
H_2O	35.625 mL	
**Total**	**50 mL**	

1X PTW (1XPBS and 0.1% Tween 20) Buffer (1L)

Component	Volume	[final]
10X Phosphate-buffered saline (PBS)	10 mL	1 X
100% Tween 20	1 mL	0.1 %
Sterile H_2O	989 mL	
**Total**	**1,000 mL**	

1X PTW/0.5% BSA Buffer (100mL)

Component	Volume	[final]
5% Bovine serum albumin (BSA)	10 mL	0.5 %
100% Tween 20	0.1 mL	0.1 %
10X PBS	10 mL	1 X
Sterile H_2O	89.9	
**Total**	**100 mL**	

1X PTW/0.2 mM Levamisole buffer (1L)

Component	Volume	[final]
100% Tween 20	1 mL	0.1 %
10X PBS	10 mL	1 X
Levamisole	0.048 g	0.2 mM
Sterile H_2O	989 mL	
**Total**	**1000 mL**	

Equipment

Dissecting microscope
Fluorescence microscope with digital camera systems
20°C Incubators
Microwave
Minicentrifuge (8,000 rpm) for microcentrifuge tubes
37°C Shaking incubator
Table-top centrifuge (1,000 rpm) for 15 mL conical tubes
Thermomixer (Possible to be maintained at 30°C and 37°C in dark environment)
